# Strategies to manage auxiliary pain medications in chronic pain trials: a topical review

**DOI:** 10.1007/s00228-022-03355-6

**Published:** 2022-07-16

**Authors:** Eivind Hasvik, Jon Michael Gran, Anne Julsrud Haugen, Lars Grøvle

**Affiliations:** 1 Department of Physical Medicine and Rehabilitation, Østfold Hospital Trust, 1714 Grålum, Norway; 2 Oslo Centre for Biostatistics and Epidemiology, Department of Biostatistics, Institute of Basic Medical Sciences, University of Oslo, Oslo, Norway; 3 Department of Rheumatology, Østfold Hospital Trust, Grålum, Norway

**Keywords:** Estimand framework, Concomitant medication, Rescue medication, Auxiliary pain medication, Randomized controlled trials, Chronic pain

## Abstract

Chronic pain trials commonly allow auxiliary pain medications such as rescue and concomitant analgesics in addition to the randomized treatment. Changes in auxiliary pain medications after randomization represent intercurrent events that may affect either the interpretation or the existence of the measurements associated with the clinical question of interest, complicating the assessment of treatment efficacy. In chronic pain trials, pain intensity typically varies and patients may take the auxiliary medications 1 day but not the next or increase and decrease the dosages temporarily while continuing their randomized study medication. This distinctive feature of auxiliary pain medications as an intercurrent event has received little attention in the literature. Further clarifications on how to manage these issues are therefore pressing. Here we provide perspectives on issues related to auxiliary pain medication-related intercurrent events in randomized controlled chronic pain trials considering the strategies suggested in the E9(R1) addendum to the ICH guideline on statistical principles for clinical trials.

## Introduction

Chronic pain trials commonly allow patients to continue their usual concomitant analgesics and many trials permit rescue medication [1]. A concomitant pain medication is defined as a pharmacological treatment started before randomization or initiated during the study and is typically used for the same indication as that of the study intervention. Rescue medications are medicines identified in the protocol that patients can take if the blinded study drug, active or placebo, fails to relieve their pain adequately. In the following text, we will use auxiliary pain medications as a collective term for rescue and concomitant pain medications. Auxiliary pain medications are non-investigational drugs that are taken in addition to, not in place of, the randomized treatment.

Any change in auxiliary pain medications after randomization and the use of rescue medication may confound or strongly affect the interpretation of estimated treatment effects. This complicates the assessment of treatment efficacy. For instance, if the experimental drug is effective, then subjects in the active drug group may reduce their concomitant pain medication or use less rescue medication than subjects in the placebo group. As a result, the difference in pain between the two groups, that is, the treatment effect, will be reduced in comparison to what one would expect to see if these supportive pain medications were not available. 

The E9(R1) addendum to the ICH guideline on statistical principles for clinical trials expands the focus on estimands, defined as a ‘precise description of the treatment effect reflecting the clinical question posed by a given clinical trial objective’ [2]. Furthermore, it provides guidance on how to treat intercurrent events (ICEs) when defining the estimand of interest. Intercurrent events are events that occur after treatment initiation and may affect either the interpretation or the existence of the measurements associated with the clinical question of interest. Discontinuation of the study intervention, or dropout, are typical intercurrent events.

In a chronic pain trial, post-randomization changes in the use of auxiliary pain medications would represent an intercurrent event [3]. For clarity, we denote auxiliary pain medication-related intercurrent events as APMICEs. Examples of APMICEs are the use of permitted rescue medication or disallowed pain medication, or a change in the dosage of an allowed concomitant pain medication. A change in dosage could be either a start or stop or a decrease or increase.

Generally, if the investigative drug was effective, then one would expect those in the active group to take less rescue medication and/or reduce their usual concomitant medication more than those in the placebo group. Due to the placebo effect, subjects in the placebo group might also reduce their concomitant medication, but probably to a lesser extent. How much each APMICE would affect the estimand would depend on the dose and the analgesic potency of the drug(s) involved. A small dose of a weak analgesic such as paracetamol will likely reduce pain less than would a large dose of a strong opioid. In addition, the drug’s duration of action and when it is taken in relation to when the outcome is measured would be of importance.

**Fig. 1:**
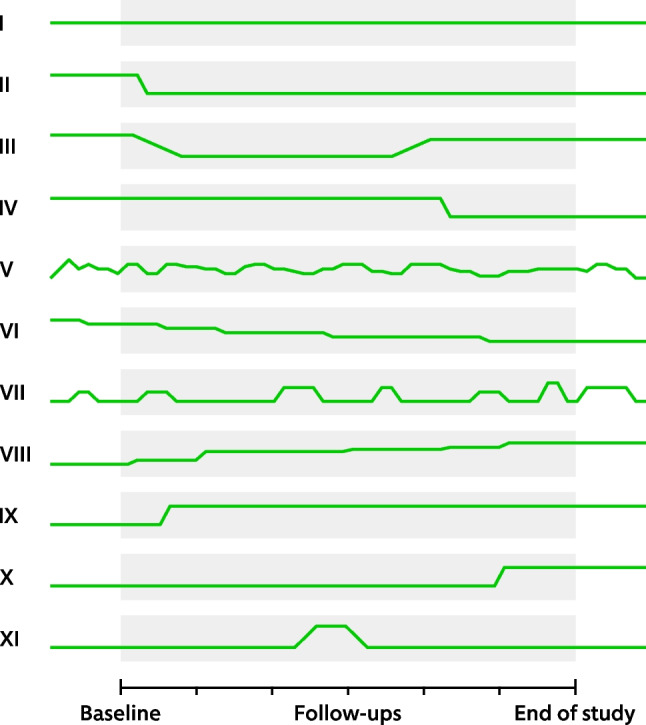
Conceivable trajectories of auxiliary pain medication use (concomitant or rescue) during a hypothetical chronic pain trial where study medication (active drug or placebo) is given from baseline (randomization) until the end of the study. The time prior to baseline could be the screening or run-in phase. Use of auxiliary pain medication could be attributable to the randomized treatment such as pain reduction due to active drug, or non-attributable, such as spontaneous improvement or deterioration over time. **Patient I**: No change. **Patient II**: Reduced early during the treatment phase. **Patient III**: Reduced early, but later increased during the treatment phase. **Patient IV:** Reduced late during the treatment phase. **Patient V**: Continued but variable use prior to randomization and throughout the study. **Patient VI**: Gradual decreased over the course of the study. **Patient VII**: Intermittent prior to and throughout the study. **Patient VIII**: Gradual increase over the course of the study. **Patient IX**: Increased early during the treatment phase. **Patient X**: Increased late during the treatment phase. **Patient XI**: Brief intermittent use

In a chronic pain trial, pain intensity typically varies from day to day and patients may take rescue medication one day but not the next or increase and decrease their concomitant medication temporarily while continuing their randomized study medication. Figure 1 illustrates conceivable trajectories of auxiliary pain medication use in a hypothetical chronic pain trial. This distinctive feature is a challenge in the management of APMICEs and has received little attention in the literature. Cai et al. [4], in a paper on estimands and missing data in clinical trials of chronic pain, briefly discussed the managing of outcomes obtained after the introduction of protocol-specified rescue medication and out-of-protocol pain treatment. Callegari et al. [5] discussed the identification and management of intercurrent events in a future phase 2 chronic pain trial. A scarcity of research and discussion on the role of auxiliary pain medication in RCTs could make it difficult for investigators to orient themselves in the field. As of yet, no published clinical trial that we are aware of explicitly planned and managed APMICEs within the estimand framework. Further clarifications on how to manage these issues are therefore pressing. The aim of this paper is to provide perspective on issues related to APMICEs in randomized controlled chronic pain trials considering the strategies suggested in E9(R1).

## The de facto estimand

In the past, the default method in chronic pain RCTs was to report the effects of the experimental drug on pain outcomes as obtained by intention-to-treat (ITT) analyses, ignoring APMICEs. This estimate, often referred to as the de facto estimand, reflects what would be the effect seen in clinical practice, that is, when auxiliary pain medications are available [6].

Previous pain trials generally gave little attention to the potential effects of auxiliary pain medication on study results. In a review of low back and neuropathic pain trials, more than one-third of the trials permitting rescue pain medication did not even report actual rescue drug consumption, and over half of the trials allowing concomitant analgesics did not report whether intake changed during the trial [1]. Some trials reported or analysed rescue medication as a secondary or explorative outcome, that is, as supplementary information to the intention-to-treat analysis. Interestingly, in the trials reporting a small or a medium effect size of the investigational drug, subjects receiving a placebo consumed 17% to 30% more rescue medication than did subjects receiving the active drug, potentially leading to underestimation of the ‘true’ underlying effects [7]. This is in line with the perception that an ITT comparison is generally conservative when participants do not fully adhere to their assigned treatment, thereby underestimating the treatment effect [6, 8]. Thus, when auxiliary pain medications are allowed but not accounted for, the de facto estimand will provide a conservative estimate of efficacy.

## The de jure estimand

The answer to the question ‘what are the benefits of this drug in itself, without the influence of auxiliary pain medications?’, is likely to be of interest to most stakeholders, including patients, clinicians, regulatory authorities and payers. Such an estimand, reflecting the ‘true’ or ‘underlying’ effect of the intervention is commonly referred to as the de jure estimand [9, 10]. Unfortunately, it is challenging to estimate the de jure estimand correctly.

**Fig. 2:**
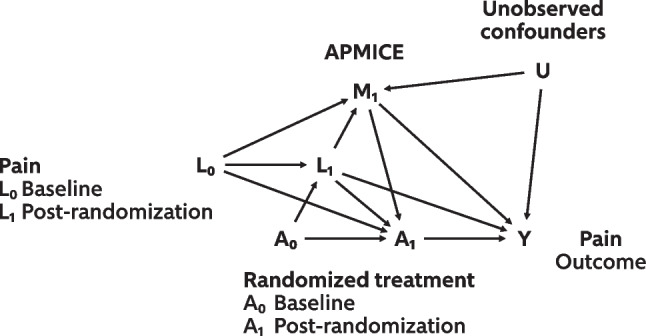
Directed acyclic graph (DAG) visualizing the causal effects of a randomized treatment (A_0_) on pain intensity at end-of-study (Y), partly going through the combination of post-randomization auxiliary medication use (M_1_) and intermediate treatment (A_1_). In this setting, auxiliary pain medication use is an intercurrent event (APMICE). The randomized treatment (A_0_) influences post-randomization pain (L_1_), which again influences later auxiliary pain medication use (M_1_), and possibly treatment adherence (A_1_). Note that post-randomization pain (L_1_) and auxiliary medication use (M_1_) here are time-dependent confounders for the effect of intermediate treatment (A_1_) on the outcome (Y). Note also that in reality, L could also include other factors than pain, such as drug-related adverse events. To estimate the de jure effect, the influence of any APMICE must be accounted for in the analysis, but simply adjusting for (conditioning on) APMICE is inappropriate. Over time, auxiliary pain medication plays both the role of a confounder, collider and a mediator. For such a time-dependent confounder, standard methods for confounding adjustment are inappropriate [15]. For example, since M_1_ is an intermediate in the chain between exposure and outcome, conditioning on it would block the effect of any past exposure on Y that goes through M_1_, resulting in over-adjustment bias [16]. Also, as M_1_ is a collider on the path A_0_ → L_1_ → M_1_ ← U → Y, adjustment for this variable would open the previously blocked path via unobserved (U) common causes of M_1_ and Y, introducing selection bias because of collider stratification [17]. The unobserved confounders (U) could, for example, be prior experience with pain medication or pain-related beliefs. To make the illustration clearer, the DAG is simplified to a setting with two time points, and certain variables, such as baseline auxiliary pain medication, are omitted

There are examples of pain trials that analysed the effect of the randomized treatment by either including the total intake of rescue medication as a covariate [11] or analysed effect modification by including rescue medication in an interaction term [12]. However, since auxiliary pain medication use can be a direct consequence of randomization, appropriate adjustment cannot be achieved by using conventional methods such as stratification, regression, or matching without introducing bias [6]. In Fig. 2, we illustrate this issue with a directed acyclic graph (DAG) visualizing the causal pathways of the randomized intervention on pain intensity at end-of-study with auxiliary medication use as an intercurrent event. Over time, auxiliary pain medication plays both the role of a confounder, collider and a mediator. For such a time-dependent confounder, standard methods for confounding adjustment are inappropriate. Unbiased estimation of the de jure effect requires the absence of unmeasured confounding for the exposure and outcome, and for the intermediate variable and outcome [13]. The unobserved confounders (U in Fig. 2) could, for example, be prior experience with pain medication or pain-related beliefs. Conditioning on non-randomized medication taken during a study is generally not recommended [14]. Poor outcomes, such as the increased pain which made auxiliary pain medication necessary, are then treated as if they are consequences of auxiliary medication, potentially leading to results indicating that the effect of auxiliary medication is harmful rather than beneficial [14].

## Strategies for managing intercurrent events due to auxiliary pain medication use

In the E9(R1), the ICH advises all trialists to specify how intercurrent events will be accounted for in the estimand, and the strategy to manage them [2]. The study objectives, the treatment regimen of interest, and potentially the perspectives of stakeholders drive the choice of strategy. Generally, following the advice of the E9(R1), intercurrent events such as the use of auxiliary pain medication should be managed as A) part of the treatment regimen, B) as an outcome or part of the outcome, or C) as a time-dependent confounder for the effect of treatment on the outcome. In the following, we will discuss how APMICEs might align with the strategies suggested in E9(R1).

## Auxiliary pain medication as part of the treatment regimen

With a *treatment-policy strategy*, the outcome data is used regardless of whether an APMICE occurs, corresponding to a de facto estimand. As shown above, this estimand reflects the effectiveness of the investigative drug in a real-world setting, that is, it shows the combined effect of the randomized treatment and any modification made by the APMICEs. If the purpose of the analysis is to estimate the effectiveness of the investigative drug in a real-world setting, or the effectiveness of a broader treatment protocol including APMICE, then a treatment-policy strategy is suitable. However, if the focus is on the effect of the active drug itself, that is, a de jure estimand, then auxiliary pain medication use should be considered as a deviation from the target intervention. In the case of non-adherence to these broader protocols, adjusting for this using the strategies outlined in the section discussing auxiliary pain medication as a confounding problem, may be relevant.

## Auxiliary pain medication as outcome

Auxiliary pain medication may be used as an outcome variable on its own or as a component of the outcome variable, that is, a composite variable. Use of rescue medication is accepted as a trial outcome by regulatory authorities [18]. Chronic pain trials commonly define rescue medication as a secondary outcome and compare rescue consumption in the randomized groups [1]. Using auxiliary pain medication consumption as an outcome can help interpret the treatment effect obtained by a treatment-policy estimand, based on the presumption that the need for auxiliary medications reflects the effect of the study drugs. However, auxiliary pain medication endpoints can be difficult to interpret. The sample size is not calculated for secondary outcomes, and it is questionable whether a typical chronic pain RCT has sufficient statistical power to analyse such endpoints. What represents clinically relevant between-group differences is not clear, and the variability of auxiliary pain medication use is generally unknown.

APMICEs could also be used to classify participants as treatment failures and estimate time-to-event outcomes, evaluating the differences between treatment groups in time to withdrawal because of unacceptable pain. However, since patients may change their auxiliary pain medication temporarily in response to a brief deterioration, the cut-off in auxiliary medication use to indicate treatment failure would need thorough consideration. Another issue is that an APMICE may be a confounding event. Ratitch [19] uses the following example:

… classifying a subject as a treatment failure because they needed rescue medication makes sense if the subject was not improving. However, if rescue was initiated because of subject’s discomfort with the possibility of placebo treatment without adequate evidence of treatment inefficacy, it would not be appropriate. The problem with defining a plausible cut-off to indicate treatment failure may be one reason why chronic pain trials seldom use APMICEs as a time-to-event outcome.

With a *composite strategy*, the endpoint of interest is modified to reflect the intercurrent event; that is, the intercurrent event is taken to be a component of the outcome variable. Composites can be constructed through summing or averaging raw or weighted scores, based on statistical modelling or expert knowledge, or by a data-driven approach, such as principal components analysis. Theoretically, this could increase domain coverage and responsiveness [20]. It could also improve statistical efficiency by addressing the multiplicity problem without requiring adjustment to the type I error [2].

Creating composite outcomes that are biologically plausible, easy to interpret and relevant to patients is complicated [21, 22], and their use in clinical trials is considered problematic [23]. There are few examples of chronic pain trials that incorporated auxiliary medications into a composite outcome, but in one trial looking at knee osteoarthritis, the responsiveness of a composite outcome consisting of pain, stiffness and function was slightly improved when the number of rescue medication pills per week was included [24].

## Auxiliary pain medication as a confounding problem

The E9(R1) addendum refers to the following strategies to manage intercurrent events as a confounding problem: (A) a *hypothetical strategy*, (B) a *while on treatment strategy*, and (C) a *principal stratum strategy.*

With a *hypothetical strategy*, we envisage a scenario in which the intercurrent event would not take place. Meaning that ‘the value of the variable to reflect the clinical question of interest is the value which the variable would have taken in the hypothetical scenario defined’ [2]. In our context, that could be, for example, to estimate what outcomes would have been in the absence of APMICE, or what outcomes would have been if APMICE were equally used in all treatment arms. Using this strategy, the intercurrent event is considered to be a confounding factor for inference about the efficacy of the intervention [19]. The objective is to estimate a treatment effect under a hypothetical scenario where time-dependent confounding does not occur. When auxiliary pain medications must be made available for ethical reasons, a treatment effect of interest might concern the outcomes if the additional medication was not available [2]. Analytical approaches for hypothetical strategies depend on the characteristics or meaning of the intercurrent event. Appropriate adjustment for post-randomization variables generally requires the use of g-methods, such as inverse probability of treatment weighting or g-computation [25, 26].

If the event implies discontinuation of study medication or dropout, then data obtained after the event can be considered as missing, and methods for dealing with missing data become applicable [9, 25]. For example, when treating auxiliary pain medication as part of the treatment regimen, as discussed earlier, instead of estimating the de facto estimand, one could censor individual’s follow-up at the time they deviated from their assigned regimen and then adjust for this artificial censoring using appropriate methods to model a situation where non-adherence did not occur [25]. This can, in principle, also be done for broader hypothetical treatment protocols, allowing for the use of APMICE in different ways.

To what extent missing techniques can be used to remove confounding due to auxiliary medication use in a chronic pain trial is largely unexplored. Pain pharmacotherapy trials generally collect detailed information about concomitant and rescue medications; however, relevant covariate data required to model hypothetical outcomes, such as measures of pain and distress immediately before an increase or decrease, are generally missing. Thus, assumptions underlying missing at random are unlikely to hold. Importantly, estimation of the hypothetical efficacy estimand relies on untestable statistical assumptions; in particular, the size of auxiliary pain medication change required to constitute an APMICE would need to be defined, and sensitivity analysis would be important [27]. To clarify the hypothetical influence of auxiliary pain medication, trialists should be encouraged to design their studies with sufficient data enabling the use of such strategies [25].

With a *while on treatment strategy*, the observation-time of interest is restricted to the time before the intercurrent event occurs. Outcomes up to the time of the event provide all necessary information about the effect of treatment. This strategy is relevant, for example, when treatment is intended to give pain relief for a terminal illness and the intercurrent event represents discontinuation of study medication. Patients with a terminal illness may discontinue a symptomatic treatment because they die, yet the success of the treatment before death is still relevant [2]. The while on treatment strategy is not appropriate when the duration of treatment is important [9], or where the intercurrent event may indicate that the study medication is no longer sufficiently effective. Thus, this strategy is generally not applicable for APMICEs and is not discussed further here.

The Initiative on Methods, Measurement, and Pain Assessment in Clinical Trials (IMMPACT) [28] suggests that ‘the effect of use of stable dosages of concomitant analgesics throughout the trial on treatment responses can be examined in the statistical analyses of the efficacy outcomes, either as a baseline covariate or with subgroup analyses comparing subjects’. This approach assumes no use of rescue medication and no changes in concomitant pain medications. The latter is difficult to confirm and unlikely to hold. An optional strategy suggested in E9(R1) is the *principal stratum strategy*, which targets subsets of the initially randomized population who would have a certain intercurrent event status, in this case APMICE, under their, potentially counterfactual, randomized treatment assignment [9]. Methods have been suggested for identification of the treatment effects in such subsets, but strong assumptions are necessary, and the usefulness of these approaches is debated [29, 30]. The principal stratum strategy has not been commonly used in clinical pharmacotherapy trials [31], and to our knowledge never for APMICEs.

## Multiple estimands

In any clinical trial, the objectives guide the choice of estimands. To put the results into a broader perspective, multiple estimands may be assessed in the same trial to manage different populations, variables, or measures of intervention effect, or to accommodate the interests of different stakeholders [32]. For instance, a de facto and different de jure estimands will answer different but complementary scientific questions. Hence, several approaches to manage APMICEs could be required in a single trial. We summarize these possible approaches in Table 1.

**Table 1:** Examples of how to manage intercurrent events related to auxiliary pain medication (APMICE) in a chronic pain trial

**Role of auxiliary pain medication**	**Strategy**	**Managing APMICEs**	**Objective**
Part of treatment regimen	*Treatment-policy*	Outcome data is used regardless of whether the APMICE occurs, comparable to the traditional intention-to-treat principle	Compare ‘active drug with auxiliary pain medication as needed’ versus ‘placebo with auxiliary pain medication as needed’
Outcome	*Composite*	The endpoint of interest is modified to reflect the APMICE	Analyse an outcome that incorporates auxiliary pain medication use with other relevant variables
	*Single*	Auxiliary pain medication use is set and analysed as an outcome	Analyse difference in auxiliary pain medication use between active and placebo
Confounder	*Hypothetical*	The APMICE is considered a confounder of the treatment effect and assumed not to have occurred	Estimate treatment effects assuming auxiliary pain medication were unavailable
	*While-on-treatment*	The observation time of interest is restricted to the time before the APMICE occurs	Compare average treatment effects until a change in auxiliary pain medication
	*Principal stratum*	Targets hypothetical subsets of the initially randomized population who have the same APMICE status regardless of the treatment arm to which they were randomized	Compare potential (counterfactual) outcomes within treated and untreated patients who always or never would take auxiliary pain medication

## Discussion

This paper discusses issues related to the analysis and interpretation of treatment effects in chronic pain trials considering the ICH E9(R1) framework of estimands and intercurrent events. The focus is on intercurrent events caused by using auxiliary pain medication, a collective term for concomitant and rescue pain medications. Depending on the purpose and context of the analysis, auxiliary pain medication may function as part of the treatment, as an outcome or as a confounder. Due to the distinctive features of APMICEs, a wide range of analytic strategies may be relevant. If the purpose is to assess the combined effect of the randomized treatment and the auxiliary pain medication, then a de facto estimand may be appropriate. If the purpose is to assess the benefits of the drug itself, without the influence of auxiliary pain medications, then a de jure estimand is appropriate.

Mallinckrodt et al. [9] suggest that when estimands are defined, intercurrent events should generally be categorized as either being part of the treatment regimen, or as ‘regimen breaking’. Those categorized as part of the regimen should be managed via a treatment-policy strategy, and those considered regimen breaking by a confounding or an outcome strategy. However, analysing APMICEs using an outcome strategy does not necessarily imply that the treatment regimen was broken. In a chronic pain trial, auxiliary pain medications are typically used in addition to, and parallel with, the study treatment. Furthermore, even in a situation where APMICEs are considered as part of a more general treatment regimen, one could still have non-adherence, and it can be of interest to adjust for this by going beyond ITT and targeting the effect that one would have seen if everyone had adhered to the treatment regimen [6].

So far, we are not aware of chronic pain trials that used confounding strategies, that is, statistical modelling to assess de jure estimands. Instead, trialists commonly limit the use of auxiliary pain medications to prevent APMICEs from influencing trial results, thereby assessing de jure estimands by trial design. Of 265 low back and neuropathic pain trials, 66% either required participants to stop or limit their use of prestudy analgesics, and more than half of the trials did not permit rescue medication. When rescue was permitted, mostly weak analgesics were allowed; of 81 neuropathic pain trials that explicitly permitted rescue medication, 59 of them only allowed paracetamol, a drug with minimal analgesic effect [1, 33]. Still, meta-analyses of these trials were based on the ITT-populations, reflecting de facto estimands [34, 35]. Results of trials that limit the use of auxiliary medications may give a better indication of the ‘true’ effect of the investigative drug, but at the cost of reduced generalizability. Estimands managing APMICEs using a de jure strategy may not necessarily reflect the situation in which chronic pain patients will find themselves in real life. Therefore, trialists need to carefully consider which drugs should be classified as part of the regimen. Another issue with a ‘deny and limit’ design is the World Medical Association’s Declaration of Helsinki, which prohibits offering patients an intervention that is less effective than the best proven one.

Very little is known about how much auxiliary pain medications affect efficacy estimates when analysed using a treatment-policy strategy. If the pain-relieving effects of each dose of the auxiliary drugs were known or believed to lie in a certain range, then the underlying ‘true’ effect on pain could be directly assigned and analysed [14]. Such exact knowledge does however not exist. The pain-relieving effects of an NSAID or a weak opioid vary according to condition and pain intensity, and efficacy estimates for acute pain cannot be extrapolated to chronic pain [36]. The Anatomical Therapeutic Chemical (ATC) classification system, which codes drugs in terms of defined daily dose (*the assumed average maintenance dose per day for a drug used for its main indication in adults*) [37] or equianalgesic dosing charts [38, 39] may be a good place to start [40].

Of the confounding strategies laid out in E9(R1), we consider the hypothetical strategy the most relevant for assessing de jure estimands in chronic pain trials, especially when the potential treatment effect of the investigational drug is not yet fully elucidated, such as in the Phase 2 setting [5]. However, a hypothetical approach would require the collection of more data, especially regarding covariates, than what is common practice [25]. Since these data are patient-reported, a hypothetical strategy will depend on motivated participants and data capturing tools that enable easy reporting outside prespecified follow-up time points. In addition, a clear definition of what represents an APMICE is needed. For instance, would taking a single tablet of rescue medication be sufficient? Callegari et al. [5] argued for not including events related to short-acting pain relief medications ‘… since it is expected that such medications are not effective for the treatment of chronic pain’. However, if the short-acting drug in question was a strong opioid and the outcome was collected while taking it, then this approach would not be appropriate. If the duration of the trial is long and the outcome is measured at the end of the study only, then the APMICEs occurring in between are likely to have little importance. Even a long-acting opioid taken for a limited period but stopped a couple of weeks prior to the end of the study may not affect the outcome. It is now standard procedure for chronic pain trials to assess outcomes repeatedly over time, which can increase the potential for auxiliary pain medications to affect the pain ratings.

Our review is limited by the lack of practical experience managing auxiliary pain medications within the estimand framework. So far, we are unaware of chronic pain trials that managed rescue or concomitant pain medications as intercurrent events and the feasibility of analysing treatment effects with APMICEs is uncertain. Experience from real-life trials, including elaboration on statistical modelling in this particular setting, is needed. We do not discuss statistical models here but note that statistical analyses depend on a specified estimand. General introductions with real-world examples from other settings exist [41].

Our use of auxiliary pain medications as a collective term for rescue and concomitant analgesics should not be confused with the definition of auxiliary medicinal products (AxMP) that was recommended by an expert group for the implementation of Regulation (EU) No 536/2014 on clinical trials [42]. That group defines AxMP as ‘a medicinal product used for the needs of a clinical trial as described in the protocol, but not as an investigational medicinal product’, which excludes concomitant medications.

## Conclusion

In chronic pain trials, post randomization changes in the use of auxiliary pain medications complicates the assessment of treatment efficacy of the investigative drug. Auxiliary pain medications may be seen as part of the treatment, as an outcome or as a confounder. In this review we discuss strategies for managing intercurrent events due to auxiliary pain medication use in light of the E9(R1) addendum to the ICH guideline on statistical principles for clinical trials.
